# Mobility of kinetochore proteins measured by FRAP analysis in living cells

**DOI:** 10.1007/s10577-021-09678-x

**Published:** 2022-01-08

**Authors:** Reito Watanabe, Yasuhiro Hirano, Masatoshi Hara, Yasushi Hiraoka, Tatsuo Fukagawa

**Affiliations:** Graduate School of Frontier Biosciences, Osaka University, Osaka, Suita 565-0871 Japan

**Keywords:** Kinetochore, CENP-C, Mis12 complex, CCAN

## Abstract

The kinetochore is essential for faithful chromosome segregation during mitosis and is assembled through dynamic processes involving numerous kinetochore proteins. Various experimental strategies have been used to understand kinetochore assembly processes. Fluorescence recovery after photobleaching (FRAP) analysis is also a useful strategy for revealing the dynamics of kinetochore assembly. In this study, we introduced fluorescence protein-tagged kinetochore protein cDNAs into each endogenous locus and performed FRAP analyses in chicken DT40 cells. Centromeric protein (CENP)-C was highly mobile in interphase, but immobile during mitosis. CENP-C mutants lacking the CENP-A-binding domain became mobile during mitosis. In contrast to CENP-C, CENP-T and CENP-H were immobile during both interphase and mitosis. The mobility of Dsn1, which is a component of the Mis12 complex and directly binds to CENP-C, depended on CENP-C mobility during mitosis. Thus, our FRAP assays provide dynamic aspects of how the kinetochore is assembled.

**Supplementary Information:**

The online version contains supplementary material available at 10.1007/s10577-021-09678-x.

## Introduction

Chromosomes harboring all the genetic information are duplicated during the S-phase and segregated into daughter cells during mitosis. Chromosome segregation is achieved via the attachment of sister chromatids to the bipolar mitotic spindle. The kinetochore, which is formed on the centromere of each sister chromatid, binds to the spindle microtubules to ensure faithful chromosome segregation (Fukagawa and Earnshaw 2014; McKinley and Cheeseman 2016; Hara and Fukagawa 2017, 2018, 2020).

The kinetochore contains numerous proteins that are divided into two major groups. One group is known as the constitutive centromere-associated network (CCAN), which consists of 16 components (centromere protein (CENP)-C, -H, -I, -K, -L, -M, -N, -O, -P, -Q, -R, -S, -T, -U, -W, and -X) and localizes to the centromere throughout the cell cycle (Okada et al. 2006; Foltz et al. 2006; Izuta et al. 2006; Hori et al. 2008; Amano et al. 2009; Nishino et al. 2012). The other group is the KMN (Knl1, Mis12, Ndc80 complexes) network, which is recruited to the CCAN during mitosis and directly binds to the spindle microtubules (McKinley and Cheeseman 2016; Pesenti et al. 2016; Nagpal and Fukagawa 2016; Hara and Fukagawa 2017; Cheeseman et al. 2006; DeLuca et al. 2006; Alushin et al. 2010; Hara and Fukagawa 2020).

CENP-C is a key CCAN component for kinetochore assembly (Saitoh et al. 1992; Fukagawa and Brown 1997; Fukagawa et al. 1999; Kwon et al. 2007; Klare et al. 2015; Weir et al. 2016). As CENP-C binds to both the CENP-A nucleosome in centromeric chromatin (Fachinetti et al. 2013; Kato et al. 2013; Falk et al. 2015; Guo et al. 2017; Watanabe et al. 2019; Ariyoshi et al. 2021) and the Mis12 complex of the KMN network at the outer kinetochores (Klare et al. 2015; Hara and Fukagawa 2017; Hara et al. 2018), CENP-C bridges the centromeric chromatin and outer kinetochore, which is associated with spindle microtubules. Because CENP-C functions as a base for kinetochore assembly, it appears to be stable in kinetochores. However, we previously showed that CENP-C dynamically changes its binding partners during cell cycle progression (Fukagawa et al. 2001; Kwon et al. 2007; Nagpal et al. 2015), suggesting that the CENP-A binding domain in the CENP-C C-terminal region is not used during interphase but rather that CENP-C binds to the CENP-A nucleosome during mitosis (Nagpal et al. 2015). We also demonstrated that CENP-C phosphorylation by cyclin-dependent kinase 1 (CDK1) facilitates the binding of CENP-C to the CENP-A nucleosome during mitosis (Watanabe et al. 2019; Ariyoshi et al. 2021).

Kinetochore dynamics have also been studied using microscopic observations. Fluorescence recovery after photobleaching (FRAP) experiments for green fluorescent protein (GFP)-tagged human kinetochore proteins revealed the dynamics of kinetochore assembly based on the exchange rates of kinetochore proteins (Hemmerich et al. 2008; Hellwig et al. 2008; Dornblut et al. 2014). FRAP analyses suggested that CENP-C is relatively dynamic during interphase compared with other CCAN proteins such as CENP-I, in human cells (Hemmerich et al. 2008). However, the protein mobility data obtained in these analyses might not be accurate, as GFP-tagged proteins were expressed transiently or their expression was controlled by exogenous promoters. Although it was difficult to generate vertebrate cells in which GFP-fused proteins were expressed under the control of an endogenous promoter more than 10 years ago, we can now use CRISPR/Cas9 genome editing, which enables the introduction of GFP-fused cDNAs into an endogenous locus. Thus, the exchange rates of proteins could be examined more accurately. Using FRAP analyses, we examined the mobility of kinetochore proteins in interphase and mitotic cells by replacing endogenous proteins with fluorescently tagged proteins that are functional at the native expression level and demonstrated various mobilities of each kinetochore component, which provides dynamic information of how the kinetochore is assembled.

## Materials and methods

### DT40 cell culture

Various chicken DT40 cells were cultured at 38.5 °C in DMEM medium (Nacalai Tesque) supplemented with 10% fetal bovine serum (FBS; Sigma), 1% chicken serum (Thermo Fisher), 10 μM 2-mercaptoethanol (Sigma), and Penicillin-Streptomycin (Thermo Fisher).

### Plasmid constructions

To express mScarlet-fused CENP-T under control of *CENP-T* endogenous promoter in DT40 cells, mScarlet-CENP-T sequence was integrated into an endogenous *CENP-T* locus using CRISPR/Cas9 genome editing. The single guide RNA (sgRNAs for CENP-T) against genomic sequence around the start codon of CENP-T was designed (Optimized CRISPR Design) (Hsu et al. 2013) and cloned into the pX330 plasmid containing SpCas9 (Addgene, 42330; pX330-CENP-T). mScarlet-CENP-T cDNA containing full length CENP-T fused with mScarlet and a drug resistance gene (*blasticidin*^*R*^ or *EcoGPT*) expressed under control of PGK promoter were inserted between ∼ 1 kb homology arms around the start site of *CENP-T* gene, using the pGEM®-T Easy Vector (mScarlet-CENP-T bs^R^ or EcoGPT).

To express mScarlet-fused CENP-H under control of *CENP-H* endogenous promoter in DT40 cells, mScarlet sequence was integrated just before stop codon of an endogenous *CENP-H* gene using CRISPR/Cas9 genome editing. The single guide RNA (sgRNAs for CENP-H) against genomic sequence around the stop codon of CENP-H was designed (Optimized CRISPR Design) (Hsu et al. 2013) and cloned into pX330 plasmid (Addgene, 42330; pX330-CENP-H). *mScarlet* and *EcoGPT* expressed under control of PGK promoter were inserted between ∼ 1 kb homology arms flanking the stop codon of *CENP-H* gene, using the pGEM®-T Easy Vector (for *CENP-H-mScarlet EcoGPT*).

To express mScarlet-fused Dsn1 under control of *Dsn1* endogenous promoter in DT40 cells, mScarlet-Dsn1 sequence was integrated into an endogenous *Dsn1* locus using CRISPR/Cas9 genome editing. The single guide RNA (sgRNAs for Dsn1) against genomic sequence around the start codon of Dsn1 was designed (Optimized CRISPR Design) (Hsu et al. 2013) and cloned into pX330 plasmid (Addgene, 42330; pX330-Dsn1). mScarlet-Dsn1 cDNA containing full-length Dsn1 fused with mScarlet and a drug resistance gene (*puro*^*R*^ or *EcoGPT*) expressed under control of PGK promoter were inserted between ∼ 1 kb homology arms flanking the start of Dsn1, using the pGEM®-T Easy Vector (*mScarlet-Dsn1 Puro*^*R*^ or *EcoGPT*).

### CRISPR/Cas9-mediated homologous recombination

Each targeting construct and the pX330 containing each sgRNA were transfected into various CENP-C-GFP cell lines (Watanabe et al. 2019), and cells containing target integrations were isolated using CRISPR/Cas9 system-mediated homologous recombination (see Plasmid constructions and Supplemental Fig. 1). Since the mScarlet-CENP-T- or mScarlet-Dsn1-targeting constructs contain drug resistance genes, the targeted cells were selected in the DT40 culture medium containing appropriate drugs.

### Genotyping PCR

DT40 cells were harvested, spun down, resuspended in 0.05 M NaOH, and heated for 10 min at 95 °C. Then, Tris-HCl (pH 8.0) was added to samples at final concentration 10% (v/v%) the solution. The genome was amplified by Tks Gflex™ DNA Polymerase (TaKaRa). PCR primers are followings: For the mScarlet-CENP-T-bs^R^ locus, forward primer CATTGCGATTGGTAGTGCAGTTTCG, reverse primer GAACTGTCTGAAGTGCTAGAGG. For the mScarlet-CENP-T-EcoGPT locus, forward primer ATATGGGCGTCGTATTCGTCCC, reverse primer GAACTGTCTGAAGTGCTAGAGG. For the CENP-H-mScarlet-EcoGPT locus, forward primer ATATGGGCGTCGTATTCGTCCC, reverse primer G AGGAGGAGCTTCACCCTTGAAGGT. For the mScarlet-Dsn1-EcoGPT locus, forward primer ATATGGGCGTCGTATTCGTCCC, reverse primer CTCTCCAGGGTCAGGTTCTGTG. For the mScarlet-Dsn1-puro^R^ locus, forward primer CTCCCCTTCTACGAGCGGCTC, reverse primer CTCTCCAGGGTCAGGTTCTGTG.

### Immunoblotting

For whole cell samples, DT40 cells were harvested, washed with PBS, and suspended in 1xLSB (Laemmli sample buffer) (final 1 × 10^4^ cells/μl), followed by sonication and heating for 5 min at 96 °C. Proteins were separated on SuperSep Ace, 5–20% (Wako) and transferred to Immobilon-P (Merck) using HorizeBLOT (ATTO). Primary antibodies used in this study were rabbit anti-chicken CENP-T (Hori et al. 2008), rabbit anti-chicken CENP-H (Fukagawa et al. 2001), rabbit anti-chicken Dsn1 (Hara et al. 2018), rabbit anti-GFP (MBL), rat anti-RFP (Chromotek), and mouse anti-α-tubulin (Sigma). Secondary antibodies were HRP-conjugated anti-rabbit IgG (Jackson ImmunoResearch), HRP-conjugated anti-mouse IgG (Jackson ImmunoResearch), and HRP-conjugated anti-rat IgG (Jackson ImmunoResearch). To increase sensitivity and specificity, Signal Enhancer Hikari (Nacalai Tesque) was used for all antibodies. The antibodies were incubated with the blotted membranes for 1 h at room temperature or for overnight at 4 °C. Proteins reacting with antibodies were detected with ECL Prime (GE Healthcare) and visualized with ChemiDoc Touch (Bio-Rad). Acquired images were processed using Image Lab 5.2.1 (Bio-Rad) and Photoshop CC (Adobe).

### Microscopy observation

DT40 cells were cytospun onto glass slides. The cells were fixed with 3% paraformaldehyde (PFA) in 250 mM HEPES-NaOH pH 7.4 for 15 min. After slides were washed with PBS and DNA was stained with 1 μg/ml DAPI in PBS for 10 min, the stained samples were washed with PBS and mounted with VECTASHIELD Mounting Medium (Vector Laboratories). Fluorescence images were acquired at 0.2-μm intervals in the *z*-axis using a Zyla 4.2 sCMOS camera (Andor) mounted on a Nikon Ti inverted microscope with an objective lens (Nikon; Plan Apo lambda 100x/1.45 NA) with a spinning disk confocal unit (CSU-W1, Yokogawa) controlled with NIS-elements (Nikon). The images in figures are the maximum intensity projection of the Z-stack generated with Fiji (Schindelin et al. 2012). Acquired images were processed using Fiji (Schindelin et al. 2012) and Photoshop CC (Adobe).

### Live cell imaging for FRAP analysis

DT40 cells were cultured at 38.5 °C for at least 2 h in DMEM medium without phenol red (Nacalai Tesque) supplemented with 10% fetal bovine serum (FBS; Sigma), 1% chicken serum (Thermo Fisher), 10 μM 2-mercaptoethanol (Sigma), Penicillin-Streptomycin (Thermo Fisher), and 0.04% (v/v%) NucSpot™ Live 650 Nuclear Stain (Biotium) to image DNA for live cell imaging (DT40 culture medium for live cell imaging). The grass bottom dish was treated by 0.5 mg/ml Concanavalin-A for 15 min at room temperature and washed by water. DT40 cells were cultured on Concanavalin-A coated glass bottom dish (IWAKI) at 37 °C for at least 15 min to attach cell to grass bottom dish. The mobilities of GFP and mScarlet-tagged kinetochore proteins and its mutants were analyzed using a confocal microscope (LSM780, Carl Zeiss) equipped with a 25× multi-immersion objective lens (NA = 0.8) to bleach the region of interest through the chromosome. Two images were collected before bleaching (approximately 0.1, 0.04 and 0.1% transmission of a 488-, 561-, and 647-nm laser, 968 ms/frame with 12-s interval, average 1256 × 128 pixels, 2.5 airy unit pinhole for three colors, 10 × zoom, 9.29 μm z-stack with 1.16-μm interval); the kinetochore signals were bleached simultaneously using 100% of a 488-nm laser, followed by the capture of a further 70 images, using the original setting. To assess the mitotic dynamics of kinetochore proteins, we treated the DT40 cells with APC/C inhibitors (25 μM of Apcin and 20 μM of proTAME) (Sackton et al. 2014) for 2 h before FRAP analysis to prevent chromosome segregation and minimize chromosome movement and arrested the cells at metaphase in mitosis. The fluorescence intensity in the bleached region was quantified using Fiji software (Schindelin et al. 2012) after projecting the z-stacks to cancel the kinetochore movement along with z-axis and subtracting background signal. An image of which the unbleached kinetochore came into the bleached region during imaging was removed from the following analysis. Photobleaching during imaging was monitored and normalized before drawing the recovery curve. The recovery curve was plotted as a relative value set the florescence intensities before and after bleaching as 1 and 0, respectively, so that the difference of the protein expression level and the bleaching efficiency between samples are normalized. The recovery curve showing mitotic dynamics of CENP-C-GFP and its mutants were fitted in Origin 8.0 (OriginLab Corp., Northampton, MA, USA) as follow:

$$I(t)={I}_0+{I}_{max}{e}^{-{k}_{off}t}$$

where *I*(*t*) is the intensity at time point *t*, *I*_0_ is the base line intensity, *I*_max_ is the maximum intensity after recovery, and *k*_off_ is recovery constant. The resident time was calculated as the inverse of *k*_off_ value.

All acquired images were processed using Fiji (Schindelin et al. 2012) and Photoshop CC (Adobe) and changed contrast that remains gamma correction.

## Results and discussion

### CENP-C is mobile independent of its CENP-A binding in interphase cells

**Fig. 1:**
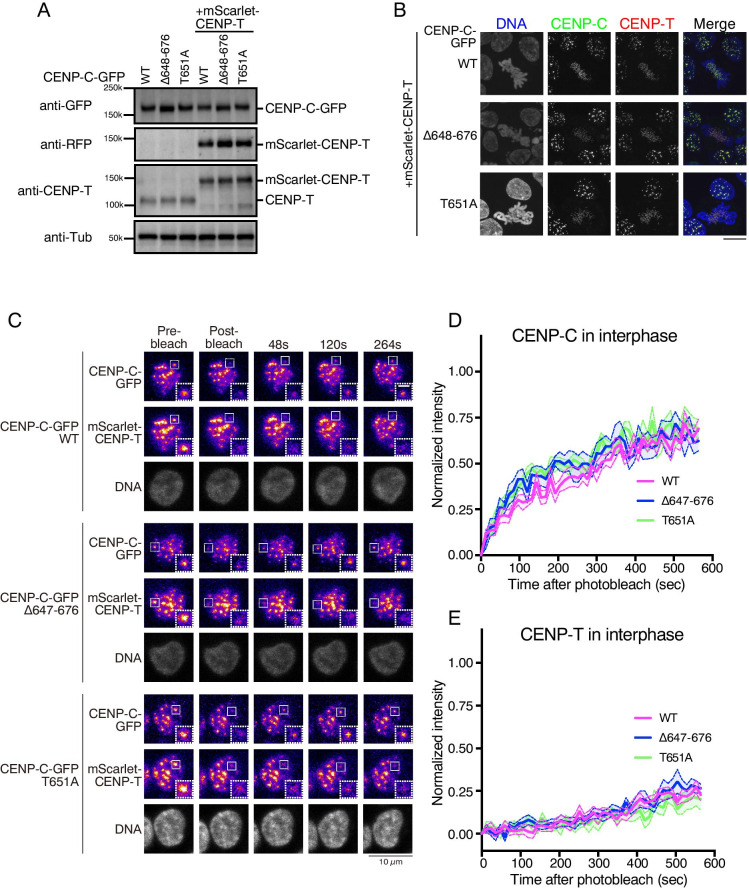
FRAP analyses of CENP-C-GFP and mScarlet-CENP-T during interphase. (**A**) Immunoblot analysis of cells in which endogenous CENP-C was replaced with GFP-fused CENP-C^WT^ (WT), CENP-C^Δ648-676^ (Δ648-676), or CENP-C^T651A^ (T651A) (left three lanes). In each cell line, as indicated in the right three lanes, mScarlet-fused CENP-T was also replaced with endogenous CENP-T. The indicated antibodies were used for immunoblot analysis. (**B**) Localization of CENP-C-GFP (green) and mScarlet CENP-T (red) in cells. DNA was stained with DAPI (blue). The scale bar indicates 10 μm. (**C**) FRAP analysis of each cell line during interphase. Indicated fluorescent proteins are shown. DNA was stained with NucSpot™ Live 650 Nuclear Stain. Left panels are images before bleaching (pre-bleach). The boxed area was bleached, and signal recovery is shown at the indicated time points. The black scale bar indicates 10 μm. The white scale bar indicates 2.5 μm for magnified panels. (**D**) Solid lines display the means of quantitative FRAP measurements for CENP-C from at least 10 interphase cells from each indicated cell line. The colored area between two dashed lines indicates the standard error for each time point. (E) FRAP results for mScarlet-CENP-T in interphase cells for each indicated cell line

To evaluate the natural molecular dynamics of kinetochore proteins in living cells, we introduced complementary DNAs (cDNAs) of kinetochore proteins fused with a fluorescent protein downstream of the endogenous promoter for each gene, using the CRISPR/Cas9 system in chicken DT40 cells. We previously generated chicken DT40 cell lines in which endogenous CENP-C was replaced with GFP-fused wild-type CENP-C (CENP-C^WT^) or CENP-C mutants: CENP-C lacking the CENP-A binding motif (CENP-C^Δ648-676^) or CENP-C bearing a mutation at the CDK1 phosphorylation site (CENP-C^T651A^), both of which do not properly bind to the CENP-A nucleosome (Watanabe et al. 2019). Using these cell lines, we introduced mScarlet-fused CENP-T cDNA downstream of the endogenous *CENP-T* promoter using the CRISPR/Cas9 system (Fig. S1A). The C-terminal tag of endogenous genes might be more suitable for our purpose than this strategy. However, we already know that the C-terminal tag of CENP-T does not work properly, because there is a histone fold domain in the CENP-T C-terminal end. Therefore, we used an N-terminal tag for CENP-T. After isolating colonies showing targeted integration of *mScarlet-CENP-T* cDNA, we confirmed that the expression of CENP-T was replaced with that of mScarlet-CENP-T by immunoblot analysis (Fig. 1A). We also confirmed the proper localization of mScarlet-CENP-T to kinetochores using fluorescence microscopy (Fig. 1B). Adequate expression and localization of CENP-C and CENP-T indicated that these cells enable us to assess the natural dynamics of CENP-C and CENP-T. We noted that the amount of mScarlet CENP-T was slightly higher than that of endogenous CENP-T (Fig. 1A). Tagged proteins might increase protein stability. Alternatively, the SV40 polyA signal sequence used for knock-In might increase mRNA stability. However, we would like to emphasize that the expression levels of mScarlet-CENP-T are appropriate to reproduce the functional kinetochores that are assembled in wild-type cells, because the growth of cells in which the essential CENP-T gene was replaced with tagged CENP-T cDNA was comparable to that of wild-type cells.

Using these lines, we performed FRAP analysis and compared the mobility of CENP-C and CENP-T at interphase (Fig. 1C–E). In each cell line, we bleached one kinetochore signal of GFP and mScarlet in interphase nuclei using a 488 nm laser and observed the recovery of fluorescent signals. We noted that mScarlet had a weak absorption spectrum at 488 nm, and a 488 nm laser bleaches both signals (Bindels et al. 2017). In cells expressing GFP-fused CENP-C^WT^, approximately 60% of GFP signals were recovered within 300 s after photobleaching (see WT in Fig. 1D). In contrast, the recovery rate of mScarlet-CENP-T signals was slower than that of CENP-C-GFP, and most mScarlet-CENP-T signals did not recover within 300 s (compare magenta curves in Fig. 1D and E), indicating that CENP-C is more mobile than CENP-T in interphase cells.

An analysis of cells expressing CENP-C^Δ648-676^ or CENP-C^T651A^ showed that the recovery rates of CENP-C^Δ648-676^ or CENP-C^T651A^ were similar to those of CENP-C^WT^ (Fig. 1C and D). CENP-T mobilities in cells expressing CENP-C^Δ648-676^ or CENP-C^T651A^ were comparable to those in cells expressing CENP-C^WT^ (Fig. 1E). The results of FRAP analyses were consistent with our previous model showing that CENP-C is not stably associated with the CENP-A nucleosome in interphase and that CENP-C is associated with the CENP-A nucleosome at mitotic kinetochores (Watanabe et al. 2019; Ariyoshi et al. 2021). We also demonstrated that the CENP-C C-terminal fragment containing the CENP-A-binding domain localizes to kinetochores only during mitosis but not to the interphase centromeres (Watanabe et al. 2019). These data suggest that centromere localization of CENP-C in interphase cells does not depend on the CENP-A-binding domain. Therefore, the CENP-A-binding domain of CENP-C does not strongly affect CENP-C mobility in interphase cells.

### CENP-C is more immobile during mitosis than interphase based on its CENP-A-binding

**Fig. 2:**
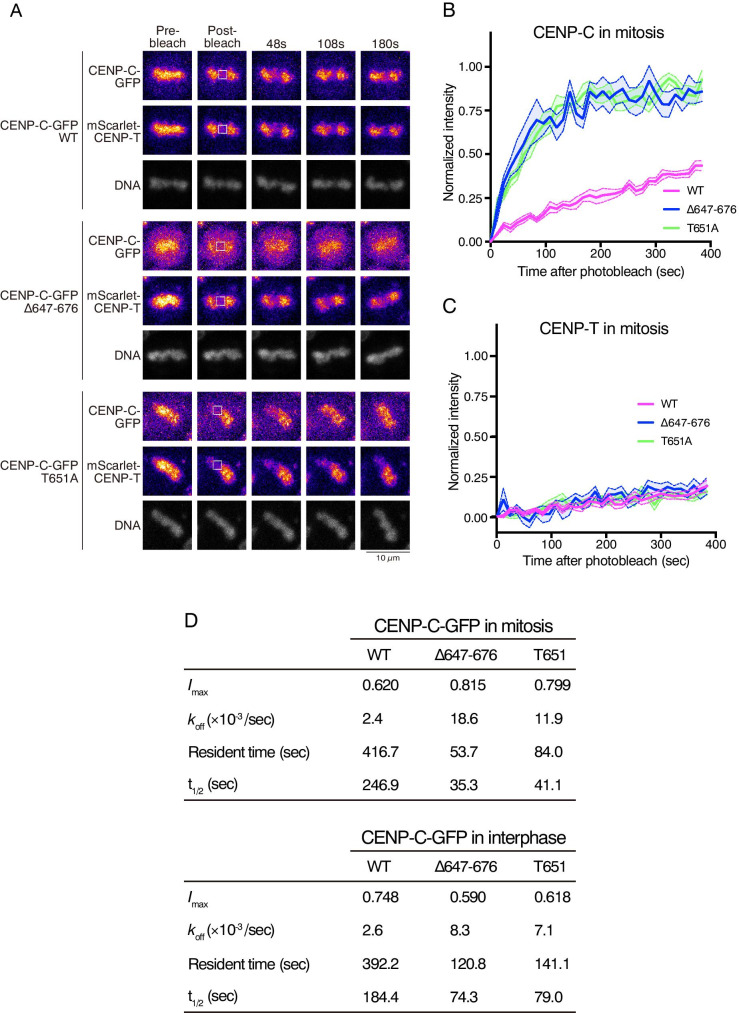
FRAP analyses of CENP-C-GFP and mScarlet-CENP-T during mitosis. (**A**) FRAP analysis of each cell line during mitosis evaluated using the indicated fluorescent proteins. DNA was stained with NucSpot™ Live 650 Nuclear Stain. Left panels are images before bleaching (pre-bleach). The boxed area was bleached, and signal recovery is shown at the indicated time points. The black scale bar indicates 10 μm. The white scale bar indicates 2.5 μm for magnified panels. (**B**) Solid lines display the means of quantitative FRAP measurements for CENP-C-GFP from at least 10 mitotic cells from each indicated cell line. The colored area between the two dashed lines indicates the standard error for each time point. (**C**) FRAP results for mScarlet-CENP-T in 10 mitotic cells from each indicated cell line. (**D**) *I*_max_ is the maximum recovery rate, t_1/2_ is the half-time of recovery, and *k*_off_ is the recovery constant. These values were calculated through curve fitting. The residence time at the kinetochores was calculated as the inverse of the *k*_off_ value. The *k*_off_ and residence time at kinetochores for CENP-C^WT^-GFP, CENP-C^Δ648-676^-GFP, and CENP-C^T651A^-GFP during mitosis and interphase are shown

Next, we examined the mobility of CENP-C in mitotic cells. We noted that the cells were treated with APC/C inhibitors to prevent chromosome segregation and minimize chromosome movement during FRAP analysis without affecting spindle attachment (see Materials and methods). Thus, we observed mitotic CENP-C mobility for a long time. In contrast to the mobility of CENP-C^WT^ in interphase cells, the recovery rate of CENP-C in mitotic cells became slow; approximately 30% of the signals were recovered within 300 s (WT in Fig. 2A and B, compared with the magenta curve in Fig. 1D), whereas the mobility of CENP-T was unchanged (Fig. 2C, compared with the magenta curve in Fig. 1E). During mitosis, CENP-C localizes to kinetochores by binding to other kinetochore proteins, including CENP-N/L (Nagpal et al. 2015), CENP-H/I/K (Klare et al. 2015), and the CENP-A nucleosome (Kato et al. 2013; Falk et al. 2016; Watanabe et al. 2019; Ariyoshi et al. 2021). The multiple binding of CENP-C to various kinetochore proteins likely contributes to its stability during mitosis.

Strikingly, the recovery rates of GFP-fused CENP-C^Δ648-676^ and CENP-C^T651A^ were faster than those of CENP-C^WT^ (Fig. 2A and B), indicating that CENP-C^Δ648-676^ and CENP-C^T651A^ are not immobilized even in mitotic cells. We previously demonstrated that the CENP-C motif region including aa 648–676 or the T651 phosphorylation of CENP-C contributes to binding to the CENP-A nucleosome during mitosis (Watanabe et al. 2019; Ariyoshi et al. 2021). These results indicate that CENP-A binding through the CENP-C motif largely contributes to the slow mobility of CENP-C during mitosis.

To extract parameters of the dynamics of CENP-C in mitosis and interphase, the recovery curves of CENP-C^WT^ and its mutants were fitted as a single exponential decay (Fig. 2D, detailed method is described in the Methods section). The residence time at the kinetochore was calculated as the inverse of the dissociation constant value (*k*_off_). We also calculated the half-time of recovery (t_1/2_) of the mobile fraction for each CENP-C during mitosis and interphase (Fig. 2D). During mitosis, the resident time of CENP-C^WT^ (more than 400 s) was approximately 5–8 times longer than that of CENP-C^Δ648-676^ and CENP-C^T651A^ mutants (50–80 s), suggesting that CENP-C^WT^ is tightly associated with the kinetochore through CENP-A binding during mitosis, which supports our previous results (Watanabe et al. 2019; Ariyoshi et al. 2021). In interphase, although recovery curves of CENP-C^Δ648-676^ and CENP-C^T651A^ mutants appeared to be similar to those of CENP-C^WT^ (Fig. 1D), resident times of mutant CENP-C (120–140 s) were shorter than those of wild type (~ 390 s). This suggests that weak CENP-C–CENP-A nucleosome interactions might occur in interphase cells. In addition, resident times of mutant CENP-C (50–80 s) during mitosis were shorter than those during interphase (120–140 s). This could be interpreted as a difference in the binding affinity of CENP-C to the CENP-H complex. Since CENP-C mutants do not bind to the CENP-A nucleosome in both interphase and mitosis, but bind to the CENP-H complex, FRAP data suggested that the interaction of CENP-H–CENP-C mutants was also decreased during mitosis, which is consistent with our previous results (Nagpal et al. 2015).

The maximum intensity after recovery (*I*_max_) indicates the ratio of the mobile and immobile molecules. Thus, we compared the *I*_max_ of CENP-C^WT^ and its mutants during mitosis and interphase. The *I*_max_ of CENP-C^Δ648-676^ and CENP-C^T651A^ mutants (0.815 and 0.799, respectively) was higher than that of WT (0.620) during mitosis (Fig. 2D). Moreover, the *I*_max_ of CENP-C^WT^ in mitosis decreased compared to that in interphase (from 0.748 to 0.620). However, the *I*_max_ of mutant CENP-C in mitosis increased compared to that in interphase (from ~ 0.6 to ~ 0.8). These results indicate that the lack of CENP-A binding of CENP-C increases the ratio of mobile molecules of CENP-C in mitosis; however, this is not the case in interphase. Together with these results, we propose that CENP-C more stably associates with centromeric chromatin via its CENP-A binding, this stable connection with centromeric chromatin is critical for linking it with outer kinetochores, because CENP-C also binds to the outer kinetochore KMN complex (Klare et al. 2015; Hara and Fukagawa 2017; Hara et al. 2018).

### CENP-H is immobilized in both interphase and mitotic cells

Our FRAP analyses showed that CENP-C is mobile in interphase and becomes immobilized during mitosis, whereas CENP-T is less mobile than CENP-C throughout the cell cycle (Figs. 1 and 2). Thus, CENP-C might not properly bind to the CENP-A nucleosome in the interphase, and the CENP-C–CENP-A interaction mainly occurs during mitosis. The mobility change in CENP-C between interphase and mitosis could be explained by the difference in the CENP-A-binding activity of CENP-C. In contrast, CENP-T is a DNA-binding protein, and DNA binding is critical for its function (Hori et al. 2008), and CENP-T must remain immobile, as observed for the centromeric histone CENP-A (Hemmerich et al. 2008). Thus, the mobility of other kinetochore proteins should be evaluated. CENP-H was selected because it is related to CENP-T (Hori et al. 2008), and CENP-H localization occurs upstream of CENP-C in interphase DT40 cells (Fukagawa et al. 2001).

**Fig. 3:**
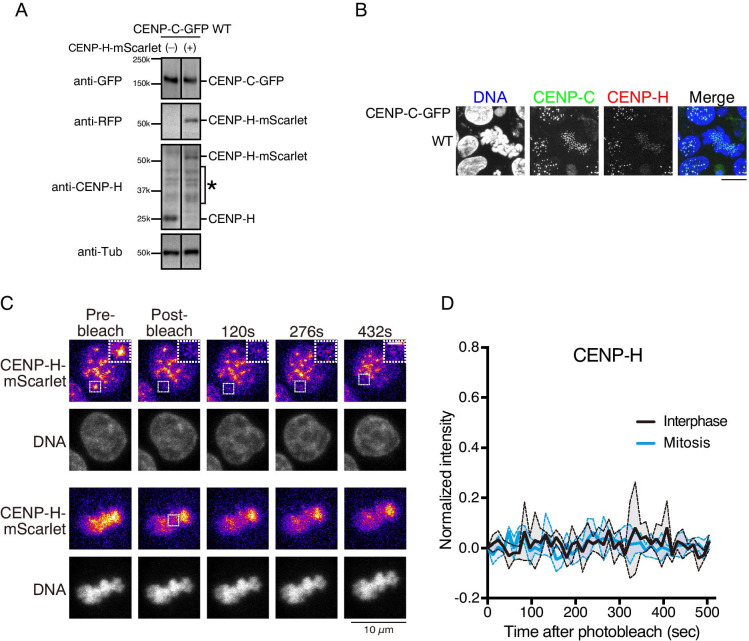
FRAP analyses of CENP-H-mScarlet during interphase and mitosis. (**A**) Immunoblot analysis of cells in which endogenous CENP-C was replaced with GFP-fused CENP-C^WT^ and endogenous CENP-H was replaced with CENP-H-mScarlet (right). In the cell line in the left lane, only CENP-C was replaced with CENP-C-GFP. The indicated antibodies were used for immunoblot analysis. An asterisk indicates non-specific bands. (**B**) Localization of CENP-C-GFP (green) and CENP-H-mScarlet (red). DNA was stained with DAPI (blue). (**C**) FRAP analysis of cells expressing CENP-H-mScarlet during interphase and mitosis. Left panels are images before bleaching. The boxed area was bleached, and signal recovery is shown at the indicated time points. The black scale bar indicates 10 μm. The white scale bar indicates 2.5 μm for magnified panels. (**D**) Solid lines display the means of quantitative FRAP measurements for CENP-H-mScarlet from at least three cells for each line. The colored area between the two dashed lines indicates the standard deviation for each time point

To test CENP-H mobility, we introduced mScarlet at the C-terminal end of the endogenous *CENP-H* locus using the CRISPR/Cas9 system in cells expressing CENP-C-GFP (Figure S1B). After confirming the expression and localization of CENP-H-mScarlet by immunoblot analysis (Fig. 3A) and microscopy (Fig. 3B), respectively, we performed FRAP analysis for CENP-H-mScarlet. As shown in Fig. 3C and D, signals of bleached CENP-H-mScarlet area were not recovered within 500 s in both interphase and mitotic cells, indicating that CENP-H, like CENP-T, was immobilized at least at 500 s.

Our FRAP data using chicken DT40 cells were slightly different from those obtained using FRAP analyses of kinetochore proteins in human cells (Hemmerich et al. 2008). A previous study of human proteins suggested that most interphase CENP-C is mobile, whereas mitotic CENP-C is immobile, which is similar to our results obtained using chicken proteins (Figs. 1 and 2); however, the dynamics of human CENP-H were different from our analyses of chicken CENP-H (Fig. 3). In human cells, most interphase CENP-H was dynamic, and most mitotic fractions were immobile, whereas chicken CENP-H was immobile throughout the cell cycle (Fig. 3).

There are at least two possible explanations for this difference. First, this difference might be related to the different structures of chicken and human kinetochores. Most components are common, although some protein organizations differ between human and chicken kinetochores. For example, CENP-C localization occurs downstream of CENP-H in chicken interphase cells (Fukagawa et al. 2001), but localization of the CENP-H-associated protein CENP-T disappeared in human CENP-C-depleted interphase cells (Watanabe et al. 2019), suggesting that CENP-H localization might occur downstream of CENP-C or the localization of CENP-C and CENP-H is interdependent in human interphase cells. Second, the difference between our chicken data and previous human data could be related to the methods used to generate cells expressing fluorescent proteins. In human studies, transient expression or random integration of GFP-fused proteins and expression levels is not the same as endogenous proteins. Excess proteins might affect the FRAP results. In contrast, we expressed proteins under control of the endogenous promoter, improving the accuracy of FRAP analyses.

### Mobility of the outer kinetochore Mis12 complex depends on CENP-C dynamics

**Fig. 4:**
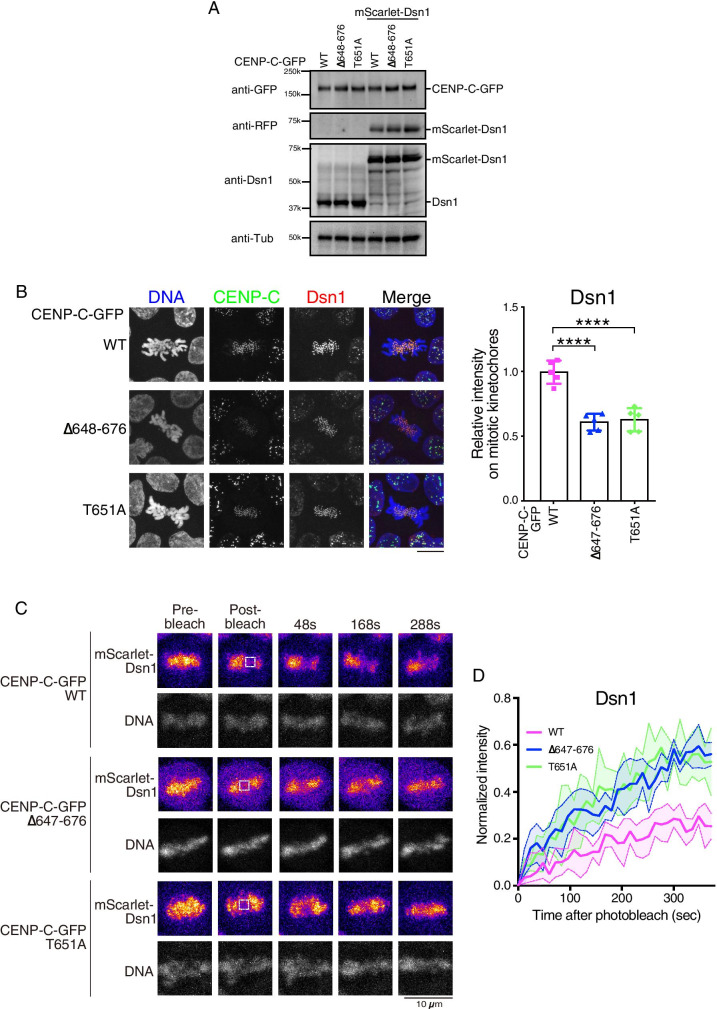
FRAP analyses of mScarlet-Dsn1 during mitosis. (**A**) Immunoblot analysis of cells in which endogenous CENP-C was replaced with GFP-fused CENP-C^WT^ (WT), CENP-C^Δ648-676^ (Δ648-676), or CENP-C^T651A^ (T651A) (left three lanes). In each cell line shown in the right three lanes, mScarlet-fused Dsn1 was also replaced with endogenous Dsn1. The indicated antibodies were used for immunoblot analyses. (**B**) Localization of CENP-C-GFP (green) and mScarlet-Dsn1 (red) in cells. DNA was stained with DAPI (blue). The scale bar indicates 10 μm. The graph shows the Dsn1 intensities at the kinetochores in each cell line. (**C**) FRAP analysis of mScarlet-Dsn1 during mitosis. Left panels are images before bleaching (pre-bleach). The boxed area was bleached, and signal recovery is shown at the indicated time points. The black scale bar indicates 10 μm. (**D**) Solid lines display the means of quantitative FRAP measurements for mScarlet-Dsn1 from at least five cells for each line. The colored area between the two dashed lines indicates the standard deviation for each time point

**Fig. 5:**
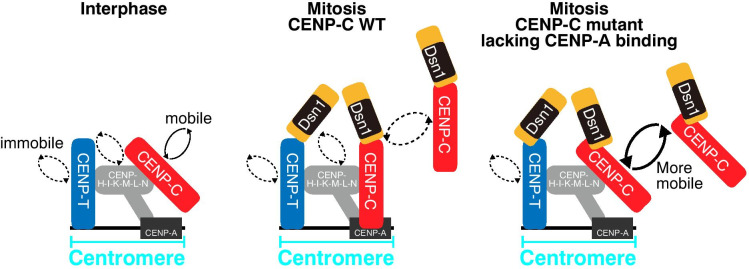
Mobility of kinetochore proteins in living cells. Schematic summary of FRAP analyses in this study. CENP-C is mobile in interphase but becomes immobile during mitosis (left two illustrations). In cells expressing CENP-C mutants lacking CENP-A binding, CENP-C is more mobile than wild-type CENP-C during mitosis; however, the mobilities of mutant CENP-C and wild-type CENP-C are comparable in interphase cells. In contrast to CENP-C, CENP-T and CENP-H are immobilized during both interphase and mitosis. CENP-T remains immobilized in mitotic cells expressing the CENP-C mutant lacking the CENP-A nucleosome-binding region (right two illustrations). Dsn1 mobility during mitosis depends on the mobility of CENP-C

Finally, we examined the mobility of the outer kinetochore Mis12 complex, which directly binds to CENP-C. We selected Dsn1, a component of the Mis12 complex, and introduced mScarlet-Dsn1 into the endogenous *Dsn1* locus (Fig. S1C) in each cell line expressing various CENP-C-GFPs (CENP-C^WT^, CENP-C^Δ648-676^, or CENP-C^T651A^). The Dsn1 C-terminal end is responsible for binding to the Ndc80 complex (Petrovic et al. 2016; Hara et al. 2018), and it is possible that the C-terminal tag loses its Ndc80C binding function. Thus, we used an N-terminal tag for Dsn1. We confirmed the expression and localization of mScarlet-Dsn1 using immunoblot analysis (Fig. 4A) and microscopy observations (Fig. 4B), respectively. Although Dsn1 levels at the kinetochores were significantly reduced in cells expressing mutant CENP-C lacking CENP-A binding (Fig. 4B), mScarlet signals were clearly detected. We previously showed that levels of CENP-C^Δ648-676^ or CENP-C^T651A^ at kinetochores were reduced, compared with those of CENP-C^WT^ (Watanabe et al. 2019), and the reduction of Dsn1 could be explained by the CENP-C reduction at the kinetochore. However, as the Dsn1-binding site is distinct from the CENP-A-binding site, the CENP-C–Dsn1 interaction should not be affected. We then performed FRAP analysis for mScarlet-Dsn1 in each cell line (Fig. 4C and D). Dsn1 starts to localize to kinetochores during G2, and kinetochore localization is maintained until the end of mitosis (Kline et al. 2006). We focused on Dsn1 mobility during mitosis. In cells expressing CENP-C^WT^, only 20% of Dsn1 was recovered within 300 s (Fig. 4C and D), indicating that Dsn1 is relatively stable in cells expressing CENP-C^WT^, which is similar to the data from a previous study on the human Mis12 complex (Hemmerich et al. 2008). In contrast, in cells expressing either CENP-C^Δ648-676^ or CENP-C^T651A^, 60% of Dsn1 was recovered in 300 s (Fig. 4C and D), indicating that more Dsn1 is mobile in cells expressing either CENP-C^Δ648-676^ or CENP-C^T651A^. CENP-C was mobilized with these mutations during mitosis (Fig. 2). Therefore, the mobility of Dsn1 depends on the mobility of CENP-C. As described, Dsn1 forms a complex with CENP-C mutants lacking CENP-A binding (CENP-C^Δ648-676^ or CENP-C^T651A^), and we suggested that Dsn1 mobility depends on CENP-C mobility during mitosis (Fig. 5).

We previously showed that deletion of the CENP-A-binding domain of CENP-C is still viable (Watanabe et al. 2019). The use of this cell line is a great advantage for analyzing the mobility of kinetochore proteins. Here, we showed that CENP-C is more mobile if the CENP-A-binding domain is deleted or mutated, and Dsn1 mobility is also changed, depending on CENP-C mobility. This might cause subtle mitotic abnormalities, although we did not observe clear mitotic defects in CENP-C-mutant cells, because KMN is still associated with centromeric chromatin via the CENP-T pathway (Watanabe et al. 2019). However, CENP-C mutants induce strong mitotic defects when the CENP-T–KMN interaction is disrupted (CENP-C pathway cells) (Hara et al. 2018; Watanabe et al. 2019). These mitotic defects could be explained by the rapid mobility of Dsn1. The mobile fraction of Dsn1 might cause unstable binding of the KMN network to microtubules. This unstable binding of KMN to microtubules can cause cell death in CENP-C pathway cells expressing mutant CENP-C but lacking CENP-A binding.

In this study, through genome editing, we introduced GFP or mScarlet-fused kinetochore-protein cDNAs into endogenous loci and performed a FRAP assay. We found that CENP-C is mobile in the interphase but becomes immobile during mitosis. CENP-C mutants lacking CENP-A binding affected the mitotic mobility of CENP-C but not its mobility during interphase. In contrast to CENP-C, CENP-T and CENP-H were immobilized during both interphase and mitosis. The mobility of Dsn1 during mitosis depends on the mobility of CENP-C (Fig. 5). Our FRAP assay provided useful information for understanding how the kinetochore is assembled.

## Supplementary Information

Fig. S1 Generation of cells in which an endogenous kinetochore protein was replaced with mScarlet-fused protein. (A) Generation of cells expressing mScarlet-CENP-T. Map of the chicken *CENP-T* locus and targeted integration of mScarlet-fused CENP-T cDNA into this allele. The indicated primers (forward and reverse) were used for polymerase chain reaction (PCR) as shown in the gel (below). PCR profiles confirming target integration of *mScarlet-CENP-T* cDNA into the *CENP-T* locus. (B) Generation of cells expressing CENP-H-mScarlet. Map of an exon encoding the stop codon for chicken CENP-H. mScarlet cDNA was introduced into this exon in frame to express CENP-H-mScarlet. The *CENP-H* gene is on the Z chromosome, and there is a single allele in DT40 cells. The indicated primers (forward and reverse) were used for PCR as shown in the gel (below). PCR profiles confirming target integration of mScarlet into the CENP-H exon. (C) Generation of cells expressing mScarlet-Dsn1. Map of chicken *Dsn1* locus and targeted integration of mScarlet-fused Dsn1 cDNA into this allele. The indicated primers (forward and reverse) were used for PCR as shown in the gel (below). PCR profiles confirming target integration of mScarlet-Dsn1 cDNA into the Dsn1 locus (PNG 444 kb)

High resolution image (EPS 3713 kb)

## Abbreviations

FRAP: Fluorescence recovery after photobleaching
CENP: Centromere protein
CCAN: Constitutive-Centromere-Associated Network
KMN: Knl1, Mis12, Ndc80 complexes
CRISPR: Clustered regularly interspaced short palindromic repeat
Cas9: CRISPR-associated protein 9

## References

[CR1] Alushin GM, Ramey VH, Pasqualato S, Ball DA, Grigorieff N, Musacchio A, Nogales E (2010). The Ndc80 kinetochore complex forms oligomeric arrays along microtubules. Nature.

[CR2] Amano M, Suzuki A, Hori T, Backer C, Okawa K, Cheeseman IM, Fukagawa T (2009). The CENP-S complex is essential for the stable assembly of outer kinetochore structure. J Cell Biol.

[CR3] Ariyoshi M, Makino F, Watanabe R, Nakagawa R, Kato T, Namba K, Arimura Y, Fujita R, Kurumizaka H, Okumura EI, Hara M, Fukagawa T (2021) Cryo-EM structure of the CENP-A nucleosome in complex with phosphorylated CENP-C. The EMBO J 40 (5):e105671. 10.15252/embj.2020105671 10.15252/embj.2020105671 PMC7917552 33463726

[CR4] Bindels DS, Haarbosch L, van Weeren L, Postma M, Wiese KE, Mastop M, Aumonier S, Gotthard G, Royant A, Hink MA, Gadella TW (2017). mScarlet: a bright monomeric red fluorescent protein for cellular imaging. Nature Methods.

[CR5] Cheeseman IM, Chappie JS, Wilson-Kubalek EM, Desai A (2006). The conserved KMN network constitutes the core microtubule-binding site of the kinetochore. Cell.

[CR6] DeLuca JG, Gall WE, Ciferri C, Cimini D, Musacchio A, Salmon ED (2006). Kinetochore microtubule dynamics and attachment stability are regulated by Hec1. Cell.

[CR7] Dornblut C, Quinn N, Monajambashi S, Prendergast L, van Vuuren C, Munch S, Deng W, Leonhardt H, Cardoso MC, Hoischen C, Diekmann S, Sullivan KF (2014). A CENP-S/X complex assembles at the centromere in S and G2 phases of the human cell cycle. Open biology.

[CR8] Fachinetti D, Folco HD, Nechemia-Arbely Y, Valente LP, Nguyen K, Wong AJ, Zhu Q, Holland AJ, Desai A, Jansen LE, Cleveland DW (2013). A two-step mechanism for epigenetic specification of centromere identity and function. Nature Cell Biol.

[CR9] Falk SJ, Guo LY, Sekulic N, Smoak EM, Mani T, Logsdon GA, Gupta K, Jansen LE, Van Duyne GD, Vinogradov SA, Lampson MA, Black BE (2015). Chromosomes. CENP-C reshapes and stabilizes CENP-A nucleosomes at the centromere. Science.

[CR10] Falk SJ, Lee J, Sekulic N, Sennett MA, Lee TH, Black BE (2016). CENP-C directs a structural transition of CENP-A nucleosomes mainly through sliding of DNA gyres. Nature Struct Mol Biol.

[CR11] Foltz DR, Jansen LE, Black BE, Bailey AO, Yates JR, Cleveland DW (2006). The human CENP-A centromeric nucleosome-associated complex. Nature Cell Biol.

[CR12] Fukagawa T, Brown WR (1997). Efficient conditional mutation of the vertebrate CENP-C gene. Human Mol Genet.

[CR13] Fukagawa T, Earnshaw WC (2014). The centromere: chromatin foundation for the kinetochore machinery. Developmental Cell.

[CR14] Fukagawa T, Pendon C, Morris J, Brown W (1999). CENP-C is necessary but not sufficient to induce formation of a functional centromere. EMBO J.

[CR15] Fukagawa T, Mikami Y, Nishihashi A, Regnier V, Haraguchi T, Hiraoka Y, Sugata N, Todokoro K, Brown W, Ikemura T (2001). CENP-H, a constitutive centromere component, is required for centromere targeting of CENP-C in vertebrate cells. The EMBO J.

[CR16] Guo LY, Allu PK, Zandarashvili L, McKinley KL, Sekulic N, Dawicki-McKenna JM, Fachinetti D, Logsdon GA, Jamiolkowski RM, Cleveland DW, Cheeseman IM, Black BE (2017). Centromeres are maintained by fastening CENP-A to DNA and directing an arginine anchor-dependent nucleosome transition. Nature Commun.

[CR17] Hara M, Fukagawa T (2017). Critical Foundation of the Kinetochore: The Constitutive Centromere-Associated Network (CCAN). Prog Mol Subcellular Biol.

[CR18] Hara M, Fukagawa T (2018). Kinetochore assembly and disassembly during mitotic entry and exit. Current Opinion Cell Biol.

[CR19] Hara M, Fukagawa T (2020). Dynamics of kinetochore structure and its regulations during mitotic progression. Cell Mol Life Sci: CMLS.

[CR20] Hara M, Ariyoshi M, Okumura EI, Hori T, Fukagawa T (2018). Multiple phosphorylations control recruitment of the KMN network onto kinetochores. Nature Cell Biol.

[CR21] Hellwig D, Munch S, Orthaus S, Hoischen C, Hemmerich P, Diekmann S (2008). Live-cell imaging reveals sustained centromere binding of CENP-T via CENP-A and CENP-B. J Biophotonics.

[CR22] Hemmerich P, Weidtkamp-Peters S, Hoischen C, Schmiedeberg L, Erliandri I, Diekmann S (2008). Dynamics of inner kinetochore assembly and maintenance in living cells. J Cell Biol.

[CR23] Hori T, Amano M, Suzuki A, Backer CB, Welburn JP, Dong Y, McEwen BF, Shang WH, Suzuki E, Okawa K, Cheeseman IM, Fukagawa T (2008). CCAN makes multiple contacts with centromeric DNA to provide distinct pathways to the outer kinetochore. Cell.

[CR24] Hsu PD, Scott DA, Weinstein JA, Ran FA, Konermann S, Agarwala V, Li Y, Fine EJ, Wu X, Shalem O, Cradick TJ, Marraffini LA, Bao G, Zhang F (2013). DNA targeting specificity of RNA-guided Cas9 nucleases. Nature Biotech.

[CR25] Izuta H, Ikeno M, Suzuki N, Tomonaga T, Nozaki N, Obuse C, Kisu Y, Goshima N, Nomura F, Nomura N, Yoda K (2006). Comprehensive analysis of the ICEN (interphase centromere complex) components enriched in the CENP-A chromatin of human cells. Genes Cells: Devoted Mol Cell Mech.

[CR26] Kato H, Jiang J, Zhou BR, Rozendaal M, Feng H, Ghirlando R, Xiao TS, Straight AF, Bai Y (2013). A conserved mechanism for centromeric nucleosome recognition by centromere protein CENP-C. Science.

[CR27] Klare K, Weir JR, Basilico F, Zimniak T, Massimiliano L, Ludwigs N, Herzog F, Musacchio A (2015). CENP-C is a blueprint for constitutive centromere-associated network assembly within human kinetochores. J Cell Biol.

[CR28] Kline SL, Cheeseman IM, Hori T, Fukagawa T, Desai A (2006). The human Mis12 complex is required for kinetochore assembly and proper chromosome segregation. J Cell Biol.

[CR29] Kwon MS, Hori T, Okada M, Fukagawa T (2007). CENP-C is involved in chromosome segregation, mitotic checkpoint function, and kinetochore assembly. Mol Biol Cell.

[CR30] McKinley KL, Cheeseman IM (2016). The molecular basis for centromere identity and function. Nat Rev Mol Cell Biol.

[CR31] Nagpal H, Fukagawa T (2016). Kinetochore assembly and function through the cell cycle. Chromosoma.

[CR32] Nagpal H, Hori T, Furukawa A, Sugase K, Osakabe A, Kurumizaka H, Fukagawa T (2015). Dynamic changes in CCAN organization through CENP-C during cell-cycle progression. Mol Biol Cell.

[CR33] Nishino T, Takeuchi K, Gascoigne KE, Suzuki A, Hori T, Oyama T, Morikawa K, Cheeseman IM, Fukagawa T (2012). CENP-T-W-S-X forms a unique centromeric chromatin structure with a histone-like fold. Cell.

[CR34] Okada M, Cheeseman IM, Hori T, Okawa K, McLeod IX, Yates JR, Desai A, Fukagawa T (2006). The CENP-H-I complex is required for the efficient incorporation of newly synthesized CENP-A into centromeres. Nat Cell Biol.

[CR35] Pesenti ME, Weir JR, Musacchio A (2016). Progress in the structural and functional characterization of kinetochores. Current Opinion Structural Biol.

[CR36] Petrovic A, Keller J, Liu Y, Overlack K, John J, Dimitrova YN, Jenni S, van Gerwen S, Stege P, Wohlgemuth S, Rombaut P, Herzog F, Harrison SC, Vetter IR, Musacchio A (2016). Structure of the MIS12 complex and molecular basis of its interaction with CENP-C at human kinetochores. Cell.

[CR37] Sackton KL, Dimova N, Zeng X, Tian W, Zhang M, Sackton TB, Meaders J, Pfaff KL, Sigoillot F, Yu H, Luo X, King RW (2014). Synergistic blockade of mitotic exit by two chemical inhibitors of the APC/C. Nature.

[CR38] Saitoh H, Tomkiel J, Cooke CA, Ratrie H, Maurer M, Rothfield NF, Earnshaw WC (1992). CENP-C, an autoantigen in scleroderma, is a component of the human inner kinetochore plate. Cell.

[CR39] Schindelin J, Arganda-Carreras I, Frise E, Kaynig V, Longair M, Pietzsch T, Preibisch S, Rueden C, Saalfeld S, Schmid B, Tinevez JY, White DJ, Hartenstein V, Eliceiri K, Tomancak P, Cardona A (2012). Fiji: an open-source platform for biological-image analysis. Nature Methods.

[CR40] Watanabe R, Hara M, Okumura EI, Herve S, Fachinetti D, Ariyoshi M, Fukagawa T (2019). CDK1-mediated CENP-C phosphorylation modulates CENP-A binding and mitotic kinetochore localization. J Cell Biol.

[CR41] Weir JR, Faesen AC, Klare K, Petrovic A, Basilico F, Fischbock J, Pentakota S, Keller J, Pesenti ME, Pan D, Vogt D, Wohlgemuth S, Herzog F, Musacchio A (2016). Insights from biochemical reconstitution into the architecture of human kinetochores. Nature.

